# Use of Animal Models to Investigate Major Allergens Associated with Food Allergy

**DOI:** 10.1155/2013/635695

**Published:** 2013-04-11

**Authors:** Jenna L. Van Gramberg, Michael J. de Veer, Robyn E. O'Hehir, Els N. T. Meeusen, Robert J. Bischof

**Affiliations:** ^1^Biotechnology Research Laboratories, Department of Physiology, Monash University, Clayton, VIC 3800, Australia; ^2^Department of Allergy, Immunology and Respiratory Medicine, Alfred Hospital and Monash University, Prahran, VIC 3181, Australia

## Abstract

Food allergy is an emerging epidemic that affects all age groups, with the highest prevalence rates being reported amongst Western countries such as the United States (US), United Kingdom (UK), and Australia. The development of animal models to test various food allergies has been beneficial in allowing more rapid and extensive investigations into the mechanisms involved in the allergic pathway, such as predicting possible triggers as well as the testing of novel treatments for food allergy. Traditionally, small animal models have been used to characterise immunological pathways, providing the foundation for the development of numerous allergy models. Larger animals also merit consideration as models for food allergy as they are thought to more closely reflect the human allergic state due to their physiology and outbred nature. This paper will discuss the use of animal models for the investigation of the major food allergens; cow's milk, hen's egg, and peanut/other tree nuts, highlight the distinguishing features of each of these models, and provide an overview of how the results from these trials have improved our understanding of these specific allergens and food allergy in general.

## 1. Introduction

In the industrialised nations, food allergy is a growing epidemic affecting all age groups and appearing at any time in life. A marked increase in the incidence of food allergy in young children is of particular concern, with a reported 6–8% of young children and 3-4% of adults having some type of food allergy [1–3]. Comparable to the trends first seen with asthma, countries such as the United States (US), United Kingdom (UK), and Australia have the highest rates of food allergy. In the past decade alone, prevalence rates in the US have increased by at least 18% [4, 5]. Similarly, a recent study in Australia found that more than 10% of a cohort of infants had challenge-proven IgE-mediated food allergy to one of the common allergenic foods (peanut, raw egg, and sesame) [6]. This escalation in the prevalence of food allergies underlies the importance of further research to improve prevention and treatment strategies.

Allergic reactions to food can range from mild responses to life-threatening anaphylaxis [7]. These aberrant allergic reactions are principally driven by a T helper type 2 (Th2) immune pathway, as evidenced by high levels of allergen-specific immunoglobulin E (IgE) [8], Th2 polarisation involving inflammatory cells, and cytokines/mediators, and the reported efficacy of therapies that inhibit Th2 immune responses in human subjects [9–12]. There is now also recognition of the innate properties of allergens and their role in Th2 polarisation of dendritic cells (DCs) and the process of allergen sensitisation [9, 13].

The most common foods that trigger food allergy are cow's milk, hen's egg, and peanuts and tree nuts, while less common food allergens include soy, wheat, fish, and shellfish [31, 32]. Food allergy is known to be most common in the first 3 years of life [1]; however, studies have shown that most food allergies that begin early in life, such as milk, egg, soy, and wheat, are generally outgrown. Conversely, allergies to peanut, tree nuts, fish, and shellfish usually persist, becoming a lifelong burden [12, 31, 33].

Animal models hold great potential as powerful tools to help answer some of the difficult questions still surrounding the food allergy epidemic. Research in humans is limited by ethical concerns and the chance of fatal anaphylactic reactions [34]. This has stimulated great interest in the use of relevant animal models to predict possible triggers for allergy, identify possible mechanisms involved in setting up the allergic pathway, as well as the testing of novel therapeutic treatments [12, 35, 36].

The purpose of this review is to discuss the application of animal models for the study of the three main food allergens: cow's milk, hen's egg, and peanuts/tree nuts and to provide an overview of the contribution of animal models to our understanding of these allergens and food allergy in general.

## 2. Small Animal Models of Food Allergy

Mice are the predominant laboratory animal used to study the development of many diseases, generally favoured for their size, short breeding cycles and manageable housekeeping, and the relative ease of genetic manipulation compared to larger models [35, 37]. The use of the murine species in research over several decades has led to the continued development of cellular and molecular tools to allow extensive investigation of mechanisms and pathways of interest. Today, mice have the most comprehensive characterisation of their biology, immunology, and genetic makeup. This background has led to mice providing the foundation for the development of numerous food allergy models. Murine models of food allergy have been investigated in several strains including C3H/HeJ, BALB/c, C57/BL6, and DBA/2 [38] (Table 1). These animals have the capacity to produce IgE and/or IgG1 anaphylactic antibodies, and strains can be divided into either high or low IgE responders [39]. One of the most challenging obstacles involved in developing murine models of food allergy is the tendency for the immune system to develop oral tolerance to ingested antigens [16, 17]. To avoid this, researchers have focussed on certain strains of mice, such as C3H/HeJ [14, 15, 23] and BALB/c [15–17, 27], which more readily display Th2 responses than other common murine strains. The use of adjuvants such as cholera toxin (CT) to help stimulate a Th2 response is also frequent in food allergy models [14–17, 22, 23, 25, 29].

The rat is another common small animal model used in studies of food allergy, with the Brown Norway (BN) strain being the most suitable for inducing specific IgE after oral sensitisation [18, 19, 40, 41]. Other rat strains such as the Wistar, Hooded Lister, and Piebald Virol Glaxo (PVG) rats, have also been examined; however, these strains fail to produce quantifiable levels of antigen-specific IgE [40]. The BN rat, as well as other murine strains, has also been used to predict the allergenicity of novel proteins, such as those used in agricultural biotechnology, as reviewed by Ladics and colleagues [42].

The guinea pig has also been used as a model to investigate the allergenicity of food proteins, specifically cow's milk (CM). Devey et al. [43] demonstrated that within 13 days of drinking CM, guinea pigs could display fatal anaphylaxis if animals were subsequently challenged. There are obstacles in translating findings from guinea pigs to the human setting, including differences in immune physiology and having to estimate IgE production in guinea pigs indirectly (e.g., through PCA) [44]. The limited knowledge and tools available to study their immune system have also led to fewer studies on this model for food allergy research, though it has been a successful model for infectious diseases [45].

## 3. Large Animal Models of Allergy

Pigs, dogs, and sheep are the main examples of large animal models that have been investigated for food allergy (Table 1). Like humans, dogs are one of the few species that can develop allergies to naturally occurring allergens including pollen, grass, house dust mite, and food [46]. Dogs have previously been used to examine other food allergens including wheat, egg, and meat proteins and displayed positive oral challenges in addition to specific IgE production, traits similar to those seen in human patients [47]. The University of California developed an atopic dog colony specifically for use in allergy research; these high IgE producing canines were first used for asthma studies involving environmental allergens such as grass pollen and ragweed, which were shown to elicit prominent airway hyperresponsiveness (AHR) in these animals [48].

Pigs (swine) represent another large animal model that presents many advantages as a comparative model for food allergy. The intestinal physiology of swine is anatomically and histologically similar to humans, with a microflora more diverse than that seen in rodent models [12, 29, 49]. Pigs also represent an outbred population, with notable variation in the quality of immune responses raised by individuals [7]. These traits are extremely important when examining the pathogenesis and immune responses to food allergens. Swine models have previously been used to investigate other allergic disorders such as asthma; in these studies, animals displayed airway obstruction, eosinophilia, and a late-phase asthmatic response following airway allergen challenge, as typically observed in human asthmatics.

Sheep have the advantage of being similar in size and physiology to humans, are placid in nature, and their use poses fewer ethical constraints compared to the use of other large animal models [37, 50]. Sheep have previously been used for allergy studies involving house dust mite (HDM) allergen with a focus on human allergic asthma [37, 51], and more recently as a model for peanut food allergy [30]. Some key advantages of using large animal models include their outbred nature, allowing studies that are more comparable to humans, the ability to conduct serial experiments within the same cohort of animals, and their relative longevity, allowing more relevant investigations into chronic disease as well as the long-term evaluation of specific therapies [37, 50].

## 4. Route of Allergen Sensitisation in Animal Models of Food Allergy

There are multiple physiological routes that can induce allergic sensitisation including oral, nasal, intraperitoneal, intragastric, and cutaneous [52]. Despite oral sensitisation being classified as one of the major routes for the sensitisation to food proteins, alternative pathways such as the skin and/or the respiratory tract may also play a role in allergic sensitisation [53]. For example, in a human study it was found that peanut sensitisation arose from environmental exposure, primarily through cutaneous or inhalation routes, rather than from maternal or infant allergen consumption [54]. Further, in a mouse model Strid et al. [55] reported that an aqueous solution of either peanut or OVA, when applied to the abraded skin induced the production of antigen-specific IgE. It is worthy to note that the most effective route of food allergen sensitisation has also been shown to vary significantly between mouse strains [15, 56]. The route of allergen sensitisation is, therefore, an important and necessary consideration for the use of any relevant animal model of food allergy and will be the topic of further discussion throughout this review.

## 5. Cow's Milk Allergy

Cow's milk (CM) allergy is one of the most prevalent food allergies that occurs in infants and young children, with the incidence estimated at 2.5% of the general population [31, 57]. CM can be divided into two main classes, whole casein (Bos d 8) which accounts for 80% of the total protein content and whey proteins that make up the remaining 20%. The casein fraction can be subdivided further into four main proteins (*α*_s1_-, *α*_s2_-, *β*-, and *κ*-casein), whilst whey contains *β*-lactoglobulin (*β*LG or Bos d 5), *α*-lactalbumin (ALA or Bos d 4), immunoglobulins (Bos d 7), bovine serum albumin (BSA or Bos d 6), and traces of lactoferrin. Although each protein has the potential to act as an allergen, casein, BLG, and ALA are believed to be the most allergenic [58–60]. Reactions to milk proteins can be either IgE mediated (usually occurring immediately or within 2 hours of ingestion) or non-IgE mediated (generally having a delayed onset). Clinical features of IgE-mediated hypersensitivities can include reactions involving the skin, respiratory tract, gastrointestinal tract, or in extreme cases, systemic anaphylactic shock [3]. The chance of developing IgE-mediated food allergy is greater in atopic humans that have the genetic predisposition towards mounting an immediate hypersensitivity reaction to food proteins. Though research has shown that most patients outgrow CM allergy by the age of 3, those that suffer from IgE-mediated hypersensitivities have a much poorer rate of outgrowing the disorder and are also at a greater risk of developing other atopic conditions [57, 61, 62]. Mice and rats have been employed in numerous studies on CM allergy (Table 1) and were first used in this field to help define the immunopathogenic mechanisms responsible for eliciting allergen-specific IgE production and other cell-mediated reactions [14, 18, 20, 23].

The Brown Norway (BN) rat is a high-immunoglobulin (specifically IgE) responder, allowing some level of comparison to atopic humans [18, 41]. In a study by Atkinson and Miller [18], sensitised BN rats displayed reaginic antibody responses to a range of milk proteins, similar to those recognised in allergic patients with CM allergy. Milk proteins were also found to be less allergenic than OVA, with the dose of antigen required to induce sensitisation being 20-fold higher. It has since been demonstrated that BN rats can also be sensitised orally and without the use of adjuvants through gavage dosing [63]. Rats sensitised in this way produced significant antigen-specific IgE responses, comparable to those seen in allergic patients [18].

Li et al. [14] used several techniques to induce IgE-mediated CM hypersensitivity in three-week-old C3H/HeJ mice. Sensitised via the intragastric (IG) route with milk proteins and CT as adjuvant, animals were boosted once a week for a five-week period. This study was one of the first murine models of CM allergy to generate systemic hypersensitivity by oral sensitisation and challenge. Six weeks after the initial sensitisation, CM-specific IgE antibody levels were significantly increased and IG challenge with allergen provoked systemic anaphylaxis, with immediate reactions regularly accompanied by respiratory symptoms. Plasma histamine levels increased significantly in CM-sensitised mice after challenge, compared with CT-sham-sensitised mice and naïve mice, suggesting histamine to be a major mediator involved in this anaphylaxis model. Heat-treated sera did not produce anaphylaxis in contrast to untreated sera, thus confirming the presence of IgE. Furthermore, cytokine production in spleen cells of allergic mice was examined, and a significant increase in the type 2 cytokines interleukin- (IL-) 4 and IL-5, but not interferon- (IFN-) *γ* was detected. This finding provided strong support that Th2 responses contribute to the development of CM allergy.

Genetic susceptibility is known to be a contributing factor towards developing food allergies; however, trying to observe the expected development of allergy in humans is practically impossible. Morafo et al. [15] aimed to investigate the susceptibility of different strains of mice to food hypersensitivity, following the sensitisation protocol Li et al. [14] first described. Their study focussed on C3H/HeJ and BALB/c mice and involved sensitising animals to both CM and peanut (PN) allergen, via the IG route with CT. Interestingly, though BALB/c mice are routinely used as models for food allergy (usually induced by systemic antigen sensitization, e.g., IP), IG sensitisation in this study failed to induce hypersensitivity reactions to either of the food allergens, whilst C3H/HeJ mice were shown to display reactions to both. CM-specific IgE levels at the time of challenge (week 6) were markedly increased in C3H/HeJ mice; however, IgE levels in BALB/c mice were only slightly higher than those of naïve mice. Furthermore, anaphylactic reactions were observed in 87% of C3H/HeJ mice, whilst none were observed in BALB/c mice or naïve mice of either strain. Anaphylactic reactions were associated with increased plasma histamine levels found only in C3H/HeJ mice. The comparison of splenocyte cytokine profiles between the two strains illustrated that in BALB/c mice IFN-*γ* production was significantly increased, whilst IL-4 and IL-10 were not. Conversely, IL-4 and IL-10, but not IFN-*γ*, levels were considerably higher in C3H/HeJ mice. Furthermore, a study by Berin et al. [24] investigated the role of TLR4 in the development of allergic sensitisation to either CM or PN proteins, in both C3H/HeJ and BALB/c mice. T-cell responses were Th2 skewed in TLR4-deficient C3H/HeJ mice but not TLR4-sufficient C3H/HeJ mice; however, this Th2 skewing was not observed in TLR4-deficient BALB/c mice. Moreover, C3H/HeJ mice were susceptible to PN-induced anaphylaxis, whilst BALB/c mice were completely resistant. This study concluded that though TLR4 status may impact T-cell responses and the severity of anaphylactic reactions to food proteins, the nature of the effect was highly dependent on the genetic background of the mouse [24]. Together, these findings suggested that genetic background plays a major role in the development of food allergies.

Other studies have demonstrated successful sensitisation to milk proteins via a systemic (IP) route [16, 17]. The most effective route of sensitisation has been shown to vary significantly between mouse strains and should be taken into consideration when developing a relevant food allergy model. A recent study by Dunkin et al. [52] assessed the impact of milk allergens via different sensitisation routes, with and without adjuvant (CT). Three-week-old C3H/HeJ mice were exposed to ALA, through IG, cutaneous, intranasal, or sublingual routes. Although sensitisation was successful via each route, cutaneous exposure was shown to induce the maximal IgE response. Interestingly, the presence of the adjuvant CT was a more significant factor for sensitisation than the actual route.

## 6. Hen's Egg Allergy

Hen's egg (HE) allergy is the second most common food allergy in children [3], with the dominant egg allergens found in egg white. Egg yolk still holds some allergenic properties; however, these are considerably lower than the four major egg white proteins, ovomucoid (OVM or Gal d 1), ovalbumin (OVA or Gal d 2), ovotransferrin (OVT or Gal d 3), and lysozyme (LYS or Gal d 4) [64]. Though OVA is the major protein in egg white (54% of total protein), OVM (Gal d 1) has been reported as the immunodominant allergen [65].

Similar to its use as a model for the study of CM allergy, the BN rat has been one of the most studied animal models for HE allergy (Table 1). Atkinson and colleagues [18, 19] effectively dosed rats orally (0.5 mL/100 g body weight) with solutions of 1.0–12.5 mg/mL OVA in distilled water twice a week, for a six-week period. In order to promote IgE, the adjuvant carrageenan was also administered IP weekly. Oral sensitisation of OVA was shown to induce both antigen-specific IgG and IgE antibodies (assessed by PCA). Levels of IgG antibody were detected in sera from day 21 onwards from animals dosed with 5 mg/mL and above, with levels peaking at day 28. Interestingly, IgE could be detected from as early as day 14 onwards, in animals given the higher doses (10.0 and 12.5 mg/mL), while the lower dose (5 mg/mL) only induced antibody from day 28 onwards. Levels of IgG were also absent at lower doses, further illustrating the impact of allergen dose on sensitisation in this model and providing potential for its use in testing factors that could either enhance or inhibit sensitisation to food proteins.

Knippels et al. [20] in subsequent studies used BN rats to further characterise the rat HE allergy model by investigating parental sensitisation to OVA without the use of adjuvants. In this study, three main factors were examined including dose (0.002–20 mg/mL), method of dose application (*ad libitum* via the drinking water or gavage), and frequency of dosing (daily, twice a week, once a week, or once every two weeks) over a period of 6 weeks. Rats were tested for anti-OVA antibodies and delayed-type hypersensitivity (DTH) responses on days 28 and 42 (separate groups). Daily administration of OVA by gavage (1 mg/rat) induced OVA-specific IgG and IgE responses in nearly all animals tested. In the same group of animals, no significant DTH response was detected at day 28 but by day 42, DTH responsiveness had developed. In comparison, upon *ad libitum *exposure to 0.002, 0.02, or 0.2 mg/mL OVA via the drinking water, no OVA-specific antibodies were produced. However, exposure to 2 or 20 mg/mL OVA caused OVA-specific IgG responses. No OVA-specific IgE was detected for either of the time-points investigated. Interestingly, the most pronounced DTH reactions were seen in rats exposed to OVA via the drinking water at day 28, with weaker responses seen by day 42. Results from this study clearly demonstrate how the method of dose application may impact on the quality and magnitude of the immune response.

In further work with this model, Knippels et al. [66] examined the effects of oral challenge with OVA in previously sensitised BN rats, reporting a minor, transient effect on breathing frequency or systolic blood pressure, similar to that observed in food allergy patients. Another study by Akiyama et al. [21] also investigated oral sensitisation in BN rats and three murine strains (BALB/c, B10A, and ASK) and found that both BN rats and B10A mice had the highest sensitisation to OVA from the models examined; this confirmed that BN rats were a suitable model for assessing the allergenicity of food proteins. This study also found that age was a contributing factor to sensitisation in BALB/c mice, with 20-week-old mice showing the highest OVA-specific IgE and IgG1 responses among the three different age groups examined (7 weeks, 20 weeks, and 1 year).

Though many studies have used OVA to examine egg allergy, OVM (or Gal d 1) has been reported as being the dominant allergen in hen's egg to cause allergic reactions in children [67]. One study conducted by Rupa et al. [22] aimed to induce allergy to OVM using a neonatal swine model. Three outbred litters of Yorkshire piglets were used for this study, where animals were sensitised IP on days 14, 21, and 35 with 100 *μ*g of crude OVM, with CT as adjuvant (10, 25, or 50 *μ*g). Pigs were fasted overnight before oral challenge on day 46 with a mixture of egg white and yoghurt. Animals were then monitored for 1 hour after challenge for symptoms of allergy. The majority of animals sensitised to OVM displayed strong skin reactivity to direct skin testing on day 35, in contrast to control pigs that did not respond. Additionally, after oral challenge, only sensitised animals showed symptoms of allergic hypersensitivity. Sera analysed from these sensitised pigs revealed OVM-specific IgG, whilst PCA reactions confirmed IgE-mediated antibody responses to OVM. Sera that were heat treated or collected from control animals failed to induce a positive PCA response. These results confirm that OVM can successfully be used to sensitise and elicit allergy in pigs, and due to their outbred nature, these animals may also provide the opportunity to investigate some of the mechanisms that underlie allergic predisposition.

## 7. Allergies to Peanuts and Tree Nuts

Despite their appearance and name, peanuts (*Arachis hypogaea*) are not actually a nut; they are a species in the legume or bean family. However, though peanuts and tree nuts originate from different families, they have both been known to contain potent allergens, with a US study reporting peanut and tree nut allergy to specifically account for 90% of fatal anaphylactic reactions [34]. Unlike other food allergies such as cow's milk and egg, PN allergy is rarely outgrown. The ubiquitous use of PN proteins, together with the apparent increase in the prevalence of PN allergy over the last few decades, has generated great attention towards research in this field [68, 69]. As many as eleven PN allergenic proteins have been categorised (Ara h 1–11) [10], with Ara h 1 and Ara h 2 classified as the major PN allergens [70, 71].

Mice have featured prominently as an animal model for PN allergy. Following from their work with a murine CM allergy model, Li et al. [23] developed a murine model of PN-induced anaphylaxis using C3H/HeJ mice-sensitised IG with a low (5 mg/mouse) or high (25 mg/mouse) PN dose together with CT as adjuvant. In this study, both doses of the allergen induced PN-specific IgE; however, the level of sensitisation was more effective with the lower dose. Systemic anaphylactic reactions after oral challenge were also more sever in mice sensitised with the low PN dose, with 12.5% of mice exhibiting fatal or near-fatal anaphylaxis. Interestingly, none of the high dose PN sensitised mice displayed such extreme reactions. Furthermore, plasma histamine levels and mast cell degranulation from ear tissue were significantly increased in sensitised mice, suggesting that histamine and other mediators released from mast cells attributed significantly to the severe reactions (including anaphylaxis) seen in the PN-sensitised mice. Investigations into T- and B-cell responses in these mice showed similarities to human patients, with allergic mice exhibiting significant *in vitro* T-cell proliferative responses to crude PN and the major allergens Ara h 1 and Ara h 2. More importantly, this study demonstrated that PN proteins are more allergenic than CM proteins, both with respect to a shorter sensitisation period (fewer doses) and the induction of hypersensitivity in adult (5-6-week-old) as well as 3-week-old mice [14, 23].

A comparison between oral and nasal routes of allergen sensitisation in a mouse model of PN allergy was performed by Fischer et al. [25], using female C57BL/6 mice, sensitised with whole PN protein extract (PPE) and CT as adjuvant. Oral sensitisation was shown to induce higher PN-specific plasma IgE antibody responses and lung eosinophilia following allergen challenge. This was in contrast to nasal sensitisation, which induced greater levels of PN-specific plasma IgG and increased airway inflammation (recruitment of macrophages) after challenge. Furthermore, only nasal sensitisation was found to favour an inflammatory response to nasal challenge with unrelated antigens. Cytokine-specific mRNA responses on whole-lung tissues were also analysed and compared for both groups before and after challenge. Before challenge, nasally and orally sensitised mice expressed similar levels of both Th1 and Th2 cytokine mRNA. After nasal challenge, however, orally sensitised mice displayed a greater increase for two Th2-associated cytokines, IL-4 and CCL-11, whereas nasally sensitised mice expressed a greater increase in the Th1 cytokine IL-17. Overall data from this study proposed that mice sensitised orally were more prone to allergic-type responses whilst nasal sensitisation was shown to promote nonallergic inflammation.

Although peanuts are not actually classified as nuts, patients allergic to peanuts also regularly develop hypersensitivity to tree nuts including almonds, Brazil nuts, cashew, and hazelnuts to name a few. Studies by de Leon et al. [72, 73] found that the major PN allergen, Ara h 2, shared similar IgE binding epitopes with allergens from almond and Brazil nuts, which may contribute to the increased rates of cosensitisation to peanuts and tree nuts in peanut-allergic individuals. Peanuts and tree nuts are frequently associated with life-threatening anaphylaxis, with both forms of allergy rarely outgrown with age. A cashew nut mouse model of allergy showed robust induction of specific IgE following transdermal sensitisation, as well as Th2 cytokines (IL-4, IL-5, and IL-13) production by cultured splenocytes from sensitised animals [26]. These sensitised mice also displayed severe systemic anaphylaxis following oral challenge with cashew nut. More recently, a long-term mouse model for hazelnut (HN) allergy was investigated to determine whether sensitivity would persist over time [27]. Findings from this study in adult BALB/c mice revealed that circulating HN-specific IgE antibodies persist for long periods (up to 8 months) despite allergen withdrawal. These long-term memory IgE responses were found to be associated with memory spleen cell IL-4 responses. This data, therefore, illustrated possible mechanisms that could be involved with persistent nut allergies, even when the allergen is withdrawn for long periods of time.

Dogs, pigs, and sheep have reportedly been used as large animal experimental models for PN allergy (Table 1). While dogs have previously been employed for the testing of numerous allergens, Teuber et al. [28] used atopic dogs for the first time to develop a canine model of PN/tree nut allergies. This study used inbred high IgE-producing spaniel/basenji dogs, subcutaneously (SC) sensitising them with commercial extracts of either 1 *μ*g of PN, English walnut or Brazil nut proteins, together with aluminium hydroxide (Alum) as adjuvant. To test allergenicity, dogs were also sensitised to soy and either wheat or barley. Intradermal skin tests, IgE immunoblotting and oral challenges were carried out with ground nut preparations. All animals skin tested at 6 months of age displayed positive wheal responses to the commercial extracts used for sensitisation, with PN generating the largest response and barley the least. IgE immunoblotting revealed specific recognition of nut proteins, with PN-sensitised dogs displaying specific IgE binding to Ara h 1, one of the major PN allergens recognised by PN-allergic subjects [70]. Within 10 minutes of oral challenge, all PN-sensitised dogs vomited and showed signs of fatigue, further demonstrating the high allergenicity to PN proteins. Overall, this study demonstrated the successful use of the dog model to display symptoms seen in food allergy sufferers, as well as a model for concurrent sensitisation to a number of food allergens.

Pigs have also been used as a large animal experimental model for PN allergy. Helm et al. [29] set out to develop a neonatal swine model of PN allergy that would not only display allergic reactions, but also immune and histological profiles similar to those seen in allergic patients. Optimal sensitisation was achieved in piglets sensitised IP with 500 *μ*g of crude PN extract with CT as adjuvant on days 9, 10, and 11 after birth, then boosted on days 18 and 25. Pigs were later challenged by either IG challenge or skin tests at 1-week intervals. Following oral challenge, physical symptoms displayed by the animals were comparable to those seen in humans, including the appearance of rashes and distress associated with the gastrointestinal (diarrhoea) and respiratory (gasping/panting) systems. Serum IgG antibodies were analysed and correlations between allergen-specific IgG levels and clinical symptom scores, suggesting that high IgG levels afford greater protection against food challenge. While IgE levels were not directly assessed in this study, repeated positive skin tests and passive cutaneous anaphylaxis indicated the presence of IgE. Finally, histological assessment of the small intestine revealed denudation of the villi, oedema, and infiltration of immune cells. Collectively, these findings demonstrate clinical, immunological, and pathological features of PN allergy as seen in humans, supporting the neonatal pig as a suitable model for investigating mechanisms of PN allergy.

More recent studies report the use of sheep as a model for PN allergy involving animals sensitised via SC injections of crude PN extract (100 *μ*g) with Alum as adjuvant [30]. Sensitisation was achieved following 3 SC injections at 2-week intervals, followed by a “boost” injection after a 1-month rest period. Concurrent SC injections of OVA (100 *μ*g) and HDM (50 *μ*g) were also given to compare allergenicity. Elevated PN-specific IgE responses were detected in 40–50% of immunised sheep, while only 10% (1 of 10 sheep) displayed detectable OVA-specific IgE. This level of sensitisation to PN allergen was similar to that seen in response to HDM allergen sensitisation here and elsewhere [51, 74] in sheep, and it likely reflects the outbred nature of this species. Though OVA was shown to have a low capacity for specific IgE induction, total OVA-specific Ig levels were shown to increase significantly. Conversely, the elevated PN-specific IgE levels were not accompanied by a notable change in PN-specific total Ig. Significantly, PN-allergic sheep showed strong IgE reactivity to two of the major peanut allergens: Ara h 1 and Ara h 2. Furthermore, 80% of sheep that responded to PN allergen with high IgE levels also displayed an immediate hypersensitivity reaction following intradermal PN challenge. The sheep model of PN allergy displays a robust systemic IgE-responsiveness to PN proteins, providing a new large animal experimental system for studies of allergen-associated immune mechanisms.

## 8. Conclusion

Despite our improved understanding of food allergy in recent years, there is still no specific therapeutic option available. Currently, strict avoidance and the use of adrenaline in the event of an accidental exposure are the only approved treatments, although several forms of immunotherapy are presently under investigation including oral (OIT), sublingual (SLIT), epicutaneous (EPIT), and subcutaneous allergen-specific immunotherapy (SCIT) [75, 76]. A recent study by Srivastava et al. [77] demonstrated that anaphylaxis in a murine model can be prevented following treatment with the Chinese herbal medicine formula FAHF-2. Findings from this study and other work from this group suggests that FAHF-2 may have the potential to treat multiple food allergies, including peanut and egg [77–79].

The high risk of anaphylaxis is a major factor limiting the development of food allergy immunotherapy in humans [75, 80]. In this context, animal models may play an important role in providing a platform for refining these treatments and ensuring thorough preclinical evaluation of their safety, prior to therapeutic human application.

## Figures and Tables

**Table 1:** Summary of the main outcomes reported from the major food allergy animal models.

Species	Strain	Sensitisation			Significant findings from the study	Refs
		Protein	Route	Adjuvant		
*Cow's milk (CM) allergy *						

	C3H/HeJ	CM (0.1, 1, or 2 mg/g body wt)	IG	CT	Increased CM-specific IgE responses, histamine levels, and type 2 cytokines; challenge provoked systemic anaphylaxis	[14]
Mouse	C3H/HeJ BALB/c	CM (1 mg/g body wt) or ground whole PN (10 mg/g body wt)	IG	CT	C3H/HeJ mice were susceptible to both CM and PN, whilst BALB/c mice were resistant	[15]
	BALB/c	CM protein, IG (0.25 *μ*g–1 mg/g body wt), or IP (10 mg)	IG, IP	±CT (oral)	Sensitisation only successful in IP-sensitised mice	[16]
	BALB/c	CM protein (1 mg/g body wt)	Oral	CT	A shorter sensitisation protocol was achieved (2 weeks) causing increased IL-4 production and a more selective IgG1 response	[17]

Rat	BN	SSM (500 *μ*g), OVA	IG	CA	Production of reaginic antibodies	[18]

*Hen's egg (HE) allergy *						

Rat	BN	OVA (0.5 mL/100 g body wt)	IG	CA (IP)	Production of reaginic antibodies and a dose response	[19]
	BN	OVA (0.002–20 mg/mL)	DW, IG	—	Method of dosing protocol greatly affected the immune responses	[20]

Rat; mouse	BN; BALB/c, B10A, and ASK	OVA (0.1 or 1.0 mg IG) or 5 mg/ml in drinking water	IG	None	BN rats and B10A mice had the highest sensitisation to OVA out of the models examined	[21]

Pig	—	Crude OVM (100 *μ*g)	IP	CT	Sensitised pigs developed wheal and flare reactions and after oral challenge displayed signs of hypersensitivity; OVM-specific IgG, IgE	[22]

*Peanut (PN) and tree nut allergy *						

	C3H/HeJ	Ground PN (5 or 25 mg)	IG	CT	Both allergen doses induced PN-specific IgE; sensitisation is more effective at 5 mg, anaphylactic reactions were also more severe	[23]
	C3H/HeJ BALB/c	Crude PN extract or CM protein (*β*LG, 1 mg)	IG	CT	C3H/HeJ mice were susceptible to PN-induced anaphylaxis, whilst BALB/c mice were completely resistant	[24]
Mouse	C57BL/6	PN protein extract (PPE; 100 ug nasal or 1 mg IG)	Nasal, IG	CT	IG sensitisation induced higher peanut- specific IgE and Th2 cytokines; nasal sensitisation caused greater IgG and IL-17	[25]
	BALB/c	Cashew nut (0.05–1 mg)	TD	None	Cashew nut-specific IgE responses; induction of Th2 cytokines (IL-4, IL-5, IL-13); oral challenge provoked systemic anaphylaxis	[26]
		Hazelnut (HN) protein (1 mg)	TD	None	HN-specific IgE persists (up to 8 months) after allergen withdrawal	[27]

Dog	—	PN, English walnut, and Brazil nut (1 *μ*g)	SC	Alum	At 6 months, intradermal skin tests were positive to nut extracts; greatest response generated by PN	[28]

Pig	—	Crude PN extract (500 *μ*g)	IP	CT	Allergy symptoms following challenge; positive skin prick test/histology from the intestine revealed villi damage and oedema	[29]

Sheep	—	Crude PN extract (100 *μ*g)	SC	Alum	40–50% of immunised sheep displayed PN-specific IgE responses; PN-allergic sheep also showed strong IgE reactivity to Ara h 1 and Ara h 2	[30]

BN: brown Norway rat; IG: intragastric; DW: drinking water; IP: intraperitoneal; TD: transdermal; SC: subcutaneous; CM: cow milk; SSM: semiskimmed milk; OVA: ovalbumin; OVM: ovomucoid; PN: peanut; CT: cholera toxin; HN: hazelnut; CA: carrageenan; Alum: aluminium hydroxide.
